# A Patient with Neonatal Cholestasis

**DOI:** 10.34763/jmotherandchild.20202404.d-20-00012

**Published:** 2021-07-16

**Authors:** Kristl G. Claeys, Luc Breysem, Eric Legius, Hilde Brems, David Cassiman, Matthieu Moisse, Pieter Vermeersch, Elena Levtchenko, Jaak Jaeken

**Affiliations:** 1 Department of Neurology, University Hospital Leuven, Leuven, Belgium; 2 Laboratory for Muscle Diseases and Neuropathies, Department of Neurosciences, KU Leuven, Leuven, Belgium; 3 Department of Radiology, KU Leuven, Leuven, Belgium; 4 Department of Human Genetics, University Hospital Leuven, Leuven, Belgium; 5 Center for Human Genetics, KU Leuven, Leuven, Belgium; 6 Department of Hepatology and Center for Metabolic Diseases, University Hospital Leuven, Leuven, Belgium; 7 Department of Neurosciences, Experimental Neurology, and Leuven Brain Institute, KU Leuven, Leuven, Belgium; 8 VIB, Center for Brain & Disease Research, Laboratory of Neurobiology, Leuven, Belgium; 9 Department of Laboratory Medicine, KU Leuven, Leuven, Belgium; 10 Center for Metabolic Diseases, University Hospital Leuven, Leuven, Belgium; 11 Pediatric Nephrology and Transplantation, University Hospital Leuven, Leuven, Belgium; 12 Department of Development and Regeneration, KU Leuven, Leuven, Belgium

**Keywords:** CAT-2, dynamin-2 deficiency, Gilbert syndrome, polyostotic fibrous dysplasia

## Abstract

The patient, a boy born in 1991, showed pronounced polyostotic fibrous dysplasia due to McCune–Albright syndrome, as well as Gilbert syndrome and Charcot–Marie–Tooth neuropathy caused by a *DNM2* mutation. In addition, the patient, his sister, mother and maternal grandfather had intermittently increased plasma arginine and lysine levels, most probably due to heterozygosity for a novel pathogenic *SLC7A2* variant.

## Patient report

**Figure 1:**
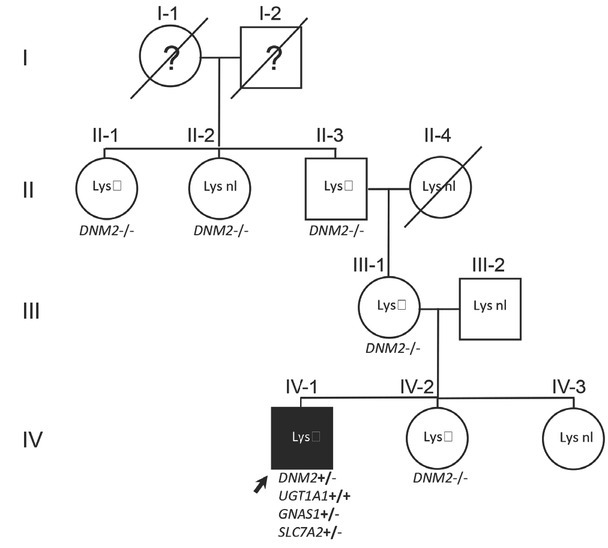
Family tree of the patient with indication of the mutations.

**Figure 2:**
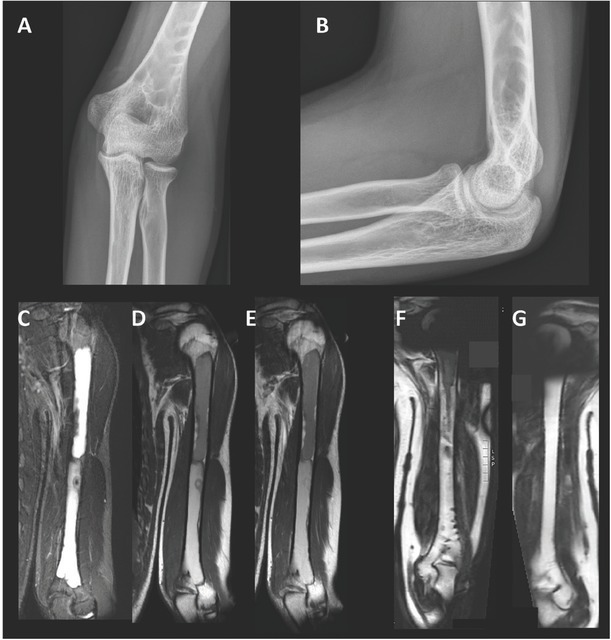
**(A, B)** Anterior–posterior and lateral X-ray of the left elbow at 17 years of age. Multiple lytic lesions are found in the medullary location of the distal humerus with thick sclerotic septae towards the cortex and a well-defined lower border (best appreciated on the anterior–posterior incidence). There is no significant cortical thinning or cortical scalloping nor periosteal reaction. Periarticular soft tissue and fat pads appear normal, no signs of hydrops; (**C, D, E)** nuclear magnetic resonance imaging (MRI) of the left humerus (17 years of age) shows abnormal heterogeneous signal intensity of the medullary cavity of the humerus shaft, hyperintense compared to the intensity of the muscle on the short tau inversion recovery (STIR) and T1-weighted (T1W) sequences. The lesion extends from the proximal to the distal end, is non-enhancing and contains septations as well as a non-specific slightly enhanced nodular lesion in the lower half of the humeral shaft. The difference in the intensities between the lower and upper halves is due to more haemorrhagic and protein-rich fluid in the upper half. These findings are compatible with a non-progressive large cystic lesion in the left humerus shaft. If there is no genetic confirmation of polyostotic fibrous dysplasia, one could consider the possibility of a lymphangioma; (**F,G)** on follow-up MRI (at 25 years of age), the lesion has decreased in size with a residual component in the proximal humerus and bony ridges in the distal humerus.

The patient, a Belgian boy, was born in 1991 after an uncomplicated 43-week pregnancy. The parents are not related. He has two healthy sisters (see Fig 1 for the family tree). His birth weight was 3,870 g, and length was 55 cm. He was admitted at 9 weeks for fluctuating jaundice since birth. Clinical examination also showed a relative microcephaly (39.7 cm [50th centile]) for a length of 62 cm (97th centile), moderate hepatosplenomegaly and dark urine. Hyperbilirubinaemia was present (13.6 mg/dL), mostly of the conjugated type (10.3 mg/dL). Liver puncture biopsy was carried out to investigate the aetiology of this cholestasis; there was hypoplasia of the internal bile ducts. Peroperative cholangiography revealed normal external bile ducts. The cholestasis progressively normalised, but since its aetiology remained unclear, a control liver puncture biopsy was performed at 1 year to evaluate possible residual damage. This showed normal bile ducts. On further follow-up, there was mild, intermittent jaundice due to genetically proven Gilbert syndrome (homozygosity for seven TA repeats in the TATA box of the promoter of the *UGT1A1* gene, as is typically associated with Gilbert syndrome: A(TA)7TAA/A(TA)7TAA). The cause of his transient cholestasis remained unclear until, after a few years, *café au lait* spots appeared on his back, raising the possibility of a McCune– Albright syndrome.^1^ This was confirmed by finding the typical c.602G<A; p.Arg201His variant (NM_000516.5) in the *GNAS1* gene in skin melanocytes. Radiological examination of the skeleton showed pronounced polyostotic fibrous dysplasia, particularly of the left humerus (Fig 2). Interestingly, in 10% of patients, liver disease is the first symptom of McCune–Albright syndrome.^2^

In addition, the patient showed mild facial dysmorphism with a slight alternating intermittent strabismus, a prominent nose bridge and mouth, a thick lower lip and high-set ears. He successfully redid his first primary school year after 1.5-years of speech therapy, and from 12 years, he started vocational training without major problems. From the age of about 9 years, bilateral pes cavus, hammer toes and tendon hyporeflexia of the lower limbs were noted. Nerve conduction studies revealed a sensorimotor axonal and demyelinating polyneuropathy. Together with a moderate neutropenia, this suggested dynamin 2 deficiency, confirmed by finding a known *de novo* pathogenic variant in *DNM2* (NM_001005360.2; c.1684_1686del; p.Lys562del).^3^ He did not have cataracts. Finally, the patient, his sister, mother and maternal grandfather showed moderate, fluctuating increases of plasma arginine (up to 200 μM; normal range: 10–130 μM) and lysine (up to 408 μM; normal range: 50–280 μM). Whole-exome sequencing showed a novel variant in *SLC7A2* (NM_001164771.2; c.1202C<T; p.Pro401Leu). Using the American College of Medical Genetics and Genomics (ACMG) classification for this variant, the categories PM2 (variant not found in healthy controls) and PP3 (conserved amino acid, with all tools predicting this variant to be pathogenic – BayesDel_addAF, DANN, DEOGEN2, EIGEN, FATHMM-MKL, M-CAP, MVP, MutationAssessor, MutationTaster, PrimateAI, REVEL and SIFT) rules are triggered, resulting in a variant of unknown significance. This is a transmembrane protein, with the variant located in the cytoplasmic exposed part of the protein. No other pathogenic mutations have been reported for this gene in the ClinVar or UniProt databases. This gene codes for a cationic amino acid transporter (CAT-2), which has been reported in a newborn in 2019.^4^ We suppose that the amino acid abnormalities in the patient and his family are an expression of heterozygosity for this defect but do not contribute to the clinical phenotype.

Actually, at the age of 29 years, the patient has a mild/ moderate ataxic gait due to his polyneuropathy with bilateral pes cavus, hammer toes and hyporeflexia of the lower limbs. In addition, he shows an alternating convergent strabismus. Otherwise, he is functioning within normal limits.
